# Improved energy efficiency using adaptive ant colony distributed intelligent based clustering in wireless sensor networks

**DOI:** 10.1038/s41598-024-55099-1

**Published:** 2024-02-22

**Authors:** K. A. Sharada, T. R. Mahesh, Saravanan chandrasekaran, R. Shashikumar, V. Vinoth Kumar, Jonnakuti Rajkumar Annand

**Affiliations:** 1 Department of Computer Science and Engineering, HKBK College of Engineering, Visvesvaraya Technological University, Bengaluru, India; 2 Department of Computer Science & Engineering, Faculty of Engineering and Technology, JAIN (Deemed-to-be University), Bengaluru, 562112 India; 3 Department of Computer Science & Engineering, SRM Institute of Science and Technology, Ramapuram campus, Chennai, Tamil Nadu 600089 India; 4 School of Electronics and Communication Engineering, REVA UNIVERSITY, Bengaluru, India; 5 School of Computer Science Engineering & Information Systems (SCORE), Vellore Institute of Technology (VIT), Vellore, India; 6 Department of Electromechanical Engineering, Arba Minch University, Sawla Campus, 4400 Arba Minch, Ethiopia

**Keywords:** Energy efficiency, Adaptive ant colony distributed intelligent based clustering algorithm (AACDIC), Spectrum sensing, Convergence time, Node power, Network capacity performance, Engineering, Energy infrastructure, Energy grids and networks

## Abstract

Optimization algorithms have come a long way in the last several decades, with the goal of reducing energy consumption and minimizing interference with primary users during data transmission over shorter distances. The adaptive ant colony distributed intelligent based clustering algorithm (AACDIC) is a key component of the cognitive radio (CR) system because of its superior performance in spectrum sensing among a group of multi-users in terms of reduced sensing errors, power conservation, and faster convergence times. This study presents the AACDIC method, which improves energy efficiency by determining the ideal cluster count using connectedness and distributed cluster-based sensing. In this study, we take into account the reality of a system with an unpredictable number of both primary users and secondary users. As a result, the proposed AACDIC method outperforms pre-existing optimization algorithms by increasing the rate at which solutions converge via the utilisation of multi-user clustered communication. Experiments show that compared to other algorithms, the AACDIC method significantly reduces node power usage by 9.646 percent. The average power of Secondary Users nodes is reduced by 24.23 percent compared to earlier versions. The AACDIC algorithm is particularly strong at reducing the Signal-to-Noise Ratio to levels as low as 2 dB, which significantly increases the likelihood of detection. When comparing AACDIC to other primary detection optimization strategies, it is clear that it has the lowest false positive rate. The proposed AACDIC algorithm optimizes network capacity performance, as shown by the results of simulations, due to its ability to solve multimodal optimization challenges. Our analysis reveals that variations in SNR significantly affect the probability of successful detection, shedding light on the intricate interplay between signal strength, noise levels, and the overall reliability of sensor data. This insight contributes to a more comprehensive understanding of the proposed scheme's performance in realistic deployment scenarios, where environmental conditions may vary dynamically. The experimental results demonstrate the effectiveness of the proposed algorithm in mitigating the identified drawback and highlight the importance of SNR considerations in optimizing detection reliability in energy-constrained WSNs.

## Introduction

Clustering in wireless sensor networks plays a vital role where sensing scheme can be fixed through a tradeoff between the performance of the sensor and the cost of the band^1^. In cognitive radio networks, low energy consumption can be achieved through adapting the sensing scheme^2^. When a sink station is far from the Cluster Head (CH), energy consumption becomes a problem where the state of every bit in the sensor is essential^3^. The optimization algorithms such as PSO are used in the simulation protocol to solve the problem and increase the reliability and efficiency of the network^4^. Clusters are formed using optimization methods, with the selection of cluster heads determined by the energy levels of the clusters. Once the shortest route has been determined, data transmission may begin^5^.

For node clustering, the authors in this study^6^, presented the firefly method, which incorporates both Partitional and hierarchical clustering. The hierarchical method compacts a large number of hierarchical clusters into a smaller set of clusters sharing common centres. There are two ways to put it into practice: the agglomerative technique, in which clusters are merged repeatedly until a single giant cluster contains all data, and the divisive approach, in which objects are divided iteratively between groups until they all belong to a single cluster. Separate data clusters are produced by Partitional clustering, but no hierarchical structure is formed. According to Ref.^7^, JFA is used at the base station to help find the best options for the status table.

This study introduces a unique clustering approach to improve the energy efficacy and durability of cognitive radio (CR) sensor networks. The emphasis is on secondary-user cooperative sensing, with the end goal being accurate sensing data that may be used to lessen the number of false alarms, increase system dependability, decrease sensing times, and increase detection rates. For the purpose of evaluation, we also use heuristic algorithms like distributed groupwise constrained clustering (DGCC), distributed global search clustering (DGSC), distributed clustering firefly groupwise Constrained (DCFGC), distributed clustering Jumper firefly groupwise constrained (DCJFGC), and adaptive ant colony distributed intelligent based clustering (AACDIC).

Nodes in a cluster will converge on the swarm particle that is closest to it in terms of neighborhood size using the DGSC algorithm^8^. The objective function is used to assess the velocities and locations of the particles, with the global optimal position serving as the termination criteria. Though it converges at about the same pace as genetic algorithms, the DGSC method is sluggish and has limited local search capabilities.

To overcome these drawbacks and effectively categorize mental nodes, DCFGC was created^9^. In the DCFGC method, brain nodes cluster quickly and efficiently by heading in the direction of the most luminous firefly. Without a status table, DCFGC will get rid of fireflies at crucial periods but will forget their history of clustering occurrences. This motivates the proposal of the DCJFGC algorithm. DCFGC and DCJFGC are non-linear optimization techniques that are inspired by the intensity and attraction of fireflies, respectively. DCJFGC compiles all historical status information and updates the status table, improving SU and PU access to the spectrum. The better detection probability and enhanced cluster communication are the results of its faster convergence speed compared to DCFGC and DGSC.

Wireless sensor networks (WSNs) play a pivotal role in monitoring and collecting data from diverse environments, ranging from industrial settings to environmental monitoring. However, the widespread deployment of WSNs is hindered by the inherent challenge of limited energy resources within sensor nodes. Prolonging the network lifetime and ensuring sustained operation are paramount concerns, particularly in scenarios where manual maintenance or replacement of nodes is impractical.

The clustering of sensor nodes, with the designation of cluster heads (CH), has emerged as a promising strategy to enhance energy efficiency by organizing nodes into hierarchies and optimizing data transmission. While existing literature has explored various clustering techniques, there remains a need for innovative approaches that can adapt to dynamic network conditions and effectively balance energy consumption across nodes.

The motivation for this study stems from the imperative to address the limitations of current clustering methods and to introduce a novel solution that leverages the synergies between adaptive ant colony optimization (ACO) and distributed intelligent clustering. ACO, inspired by the foraging behavior of ants, offers a decentralized optimization paradigm that aligns with the self-organizing principles inherent in WSNs. The integration of distributed intelligence further augments the adaptability of the system to dynamic changes in the network, providing a comprehensive solution to the challenges faced by conventional clustering techniques.

By undertaking this research, we aim to contribute to the overarching goal of extending the operational lifespan of WSNs, thereby facilitating their widespread and sustainable deployment. The study endeavors to not only demonstrate the efficacy of the proposed method in simulation environments but also to explore its practical applicability and scalability in real-world scenarios. The findings are anticipated to have implications for diverse fields, including environmental monitoring, industrial automation, and smart infrastructure, where the efficient utilization of WSNs is pivotal for informed decision-making and resource optimization.

This study makes a significant contribution to the literature on energy-efficient clustering in wireless sensor networks (WSNs) by introducing an innovative approach that combines adaptive ant colony optimization (ACO) with distributed intelligent clustering. The integration of ACO leverages the collective behavior of ants to optimize cluster formation, routing paths, and data aggregation, thereby addressing the energy efficiency challenges inherent in WSNs. The inclusion of distributed intelligence further enhances the adaptability of the system to dynamic network conditions.

Compared to existing studies, which often focus on specific clustering methods or dynamic adaptations, our approach provides a unique synthesis of nature-inspired optimization and intelligent clustering. The study aims to demonstrate not only the efficacy of the proposed method in terms of energy savings but also its adaptability to varying network topologies and conditions.

Moreover, the research extends beyond simulation-based evaluations, incorporating considerations for real-world deployment challenges and scalability. By addressing the limitations identified in the literature and introducing a comprehensive solution, this study endeavors to contribute valuable insights and practical implications for the design and implementation of energy-efficient WSNs. The findings are anticipated to advance the understanding of clustering strategies in sensor networks and provide a foundation for future research in the domain of intelligent and adaptive energy optimization.

Overall, the primary goal of AACDIC method is to shorten the average sensing time for PUs by enlisting the help of SUs working in tandem. The method uses objective functions to determine the strength of the light force, therefore dividing the population into smaller sub-swarms. Several performance metrics are used to compare the effectiveness of different clustering algorithms, such as DGSC, DCFGC, DCJFGC, and AACDIC. These metrics include conservative merging duration, cluster node power consumption, PU/SU power consumption, and spectrum sensing detection techniques.

This paper is further organized as follows: In Sect. “Background”, we cover the latest findings and difficulties in studying cognitive radio networks. In Sect. “Methodology”, we summarize how the suggested AACDIC algorithm might help lower cluster communication power. The suggested algorithm's findings and comments are shown in Sect. “Results and discussions”, while the paper’s conclusions and suggestions for further study are presented in Sect. “Conclusions”.

## Background

To simplify spectrum sensing in cognitive radio networks, this study^10^ proposed the DGCC method. There are three phases to a DGCC operation: detecting the channel, transmitting a beacon, and coordinating the two. In the channel sensing phase, the collected sensing data is used to locate and evaluate specific open channels. In the beaconing phase, nodes announce themselves across the available channels. The intensity of neighbouring beacon signals is then calculated via intra-cluster coordination, and pairwise distances are announced. Clusters and cluster leaders are then updated as part of the inter-cluster coordination phase (CHs). The control overhead caused by changes in the network topology is enhanced whenever Secondary Users (SUs) or Primary Users (PUs) switch roles.

- The SPDA channel allocation strategy for SUs in cognitive radio networks was suggested by Nair et al.^11^, which makes use of a Gale-Shapley matching algorithm. To improve secondary network performance and reduce main network congestion, SPDA takes into account both SU class of service standards and channel quality when assigning channels. However, it does not include PU or other measures of SU satisfaction and concentrates solely on SU satisfaction.
- Statistical spectrum status information over numerous time slots was used to investigate the average spectrum efficiency for multi-radio multi-hop cognitive radio networks (CRN)^12^. Using a -iteration convex optimization approach, they devised the Multi-step convex approximation (SMCA) scheme to simplify the difficult fractional Mixed-Integer Nonlinear Programming (MINLP) feature into a sequence of consecutive convex optimizations. However, data transmission in cognitive radio networks becomes somewhat more complicated due to SMCA.
- To assess the value of non-switching spectrum handoff approaches, especially in multi-class SUs, Shakeel et al.^13^ introduced a Markov-based analytical model. Their solution prioritizes traffic for enhanced quality of service (QoS) in delay-sensitive applications by combining underlay and interweave spectrum access techniques in cognitive radio networks. When main traffic volumes are high, however, SUs may have to wait longer for availability on each channel.
- Semi-tensor product compressed spectrum sensing (STP-CSS) was first proposed by Fang et al.^14^, which uses semi-tensor product methods to compress the energy required to reconstruct a spectrum. By using a series of wideband random filters, the STP-CSS method was developed, which speeds up spectrum sensing through parallel perceptual reconstruction. Despite the lower compression ratios, this method increased energy consumption inside the CRN.
- With the goal of achieving a compromise between bandwidth utilisation and sensing performance, Y. Mi et al.^15^ proposed a semi-soft decision scheme for CSS. Their methodology is split into three parts: regional detection, information reconstruction, and worldwide decision-making. While this technique improves sensing performance, it increases the cognitive radio network's bandwidth needs, especially when several SUs send data to the fusion centre.

Based on experimental findings, the authors in these studies^16–20^ developed a heuristic approach to identify appropriate sensing and transmission lengths, resulting in a good compromise between throughput and energy consumption for secondary users. The Table 1 presents the advantages and drawbacks of existing work.

**Table 1:** Advantages and drawbacks of Existing work.

Protocol	Advantages	Drawbacks
LEACH	Introduced data fusion to reduce the redundant data More energy efficient than direct transmission approach It uses TDMA schedule to avoid unnecessary collision among CHs	The randomness in LEACH may result in large number of CHS from the optimal count There is no criterion for optimal CH selection CHs directly send data to base station
HEED	It supports uniform and non uniform node distribution It provides balanced clusters and uniform distribution of CHs HEED improves energy efficiency and network scalability through inter cluster communication in multi hop way	Complexity of algorithm increases due to repeated iterations to form clusters The cluster head near to BS consumed more power than any other node due to multihop communication that may cause quick loss of energy Unbalanced energy consumption due to higher number CHs produced than the expected number of CHs
PEGASIS	Energy-efficient due to the data aggregation approach. Reduces the number of transmissions by passing data through neighboring nodes. Suitable for linear topologies	Limited scalability for non-linear network topologies. Susceptible to node failures as the failure of one node affects the entire chain. Requires synchronization among nodes for efficient data aggregation
BCDCP	Balanced energy consumption through a dynamic clustering process. Uses centralized control for cluster head selection. Minimizes energy consumption during data transmission	High communication overhead during the clustering process. Sensitive to initial energy levels of nodes, affecting cluster formation. Scalability challenges for large-scale networks
TEEN	Adaptive to network changes through dynamic threshold adjustments. Efficient in handling event-based data gathering. Low latency for event detection and reporting	Susceptible to false positives due to dynamic threshold adjustments. Limited scalability for large-scale networks. Increased communication overhead during threshold adjustments
SEP	It works on hierarchical clusters It provides balanced cluster size and TDMA utilized for intra cluster transmission	It has large control message overhead with respect to other clustering techniques The total energy consumption may increase due to multi hop intra-cluster communication

1. HEEL: A new clustering method to improve wireless sensor network lifetime:
  The HEEL method introduces a novel clustering approach aimed at enhancing the lifetime of Wireless Sensor Networks. It is essential to assess how HEEL achieves improved energy efficiency and whether its principles align with the goals of the current research.
2. DCSE: A dynamic clustering for saving energy in wireless sensor network:
  The DCSE approach focuses on dynamic clustering to conserve energy in WSNs. Understanding the dynamic aspects of clustering and the strategies employed by DCSE can offer valuable insights into alternative methodologies for achieving energy savings in sensor networks.
3. Designing a dynamic protocol for real-time industrial internet of things-based applications by efficient management of system resources:
  This study emphasizes the design of a dynamic protocol tailored for real-time industrial internet of things (IIoT) applications. Investigating how this protocol efficiently manages system resources, especially in the context of CH, can contribute to a comprehensive understanding of energy-efficient WSNs.

In comparison to the aforementioned studies, the current research leveraging adaptive ant colony distributed Intelligent Clustering brings a unique perspective. While the reviewed studies focus on specific clustering techniques and dynamic adaptations, the integration of ACO and distributed intelligence provides an alternative mechanism for optimizing energy efficiency in Wireless Sensor Networks. Subsequent sections will delve into the specific methodologies and contributions of these studies, allowing for a comprehensive evaluation of the diverse approaches employed in the literature.

Although these studies have made important contributions, they also have drawbacks such as high overhead, excessive energy consumption, latency problems, and insufficient satisfaction between SUs and PUs. The Adaptive Ant Colony Distributed Intelligent based Clustering (AACDIC) method was designed to increase convergence speed and detection performance in order to overcome these restrictions.

## Methodology

**Figure 1:**
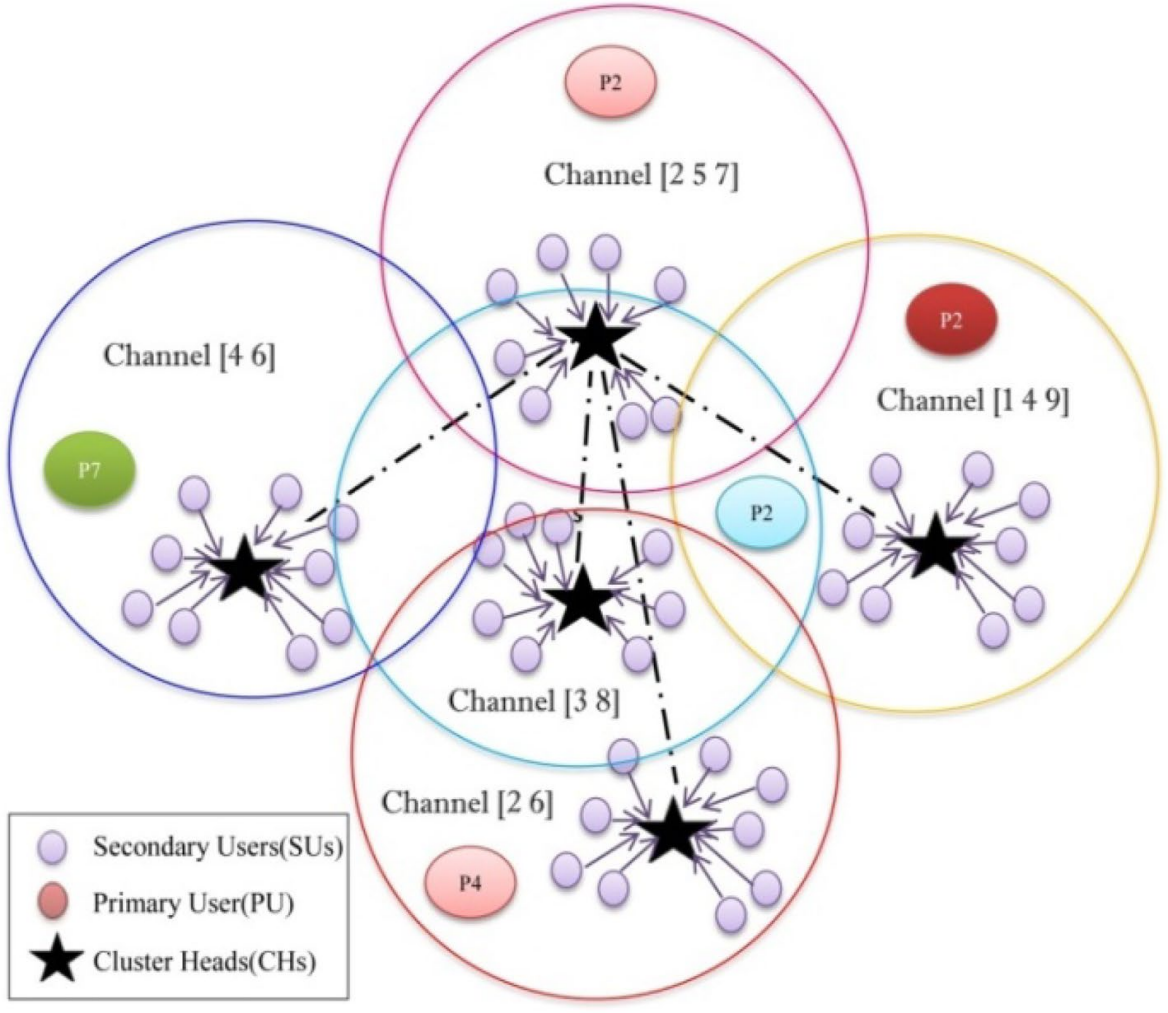
An illustration for cooperative AACDIC clustering structure.

In Fig. 1, we present the unique AACDIC approach, which functions in the cognitive radio sensor networks' dynamic spectrum environment (CRSNs). Each CRSN node actively searches for open channels within this framework, which is partitioned into clusters according to the degree of connectedness between nodes (denoted by the letter *'K*'). Frequency bands are assigned to primary users (PUs) and secondary users (SUs) in a continuous and consecutive manner. Within the CRSN, nodes are intelligently clustered utilising swarm intelligence incorporated in the clustering process, thereby mitigating the persistent re-clustering problem that plagues conventional approaches. In this sensor network, several nodes coexist, and Eq. (1) is used to find the best possible clustering of the nodes.

1 $${K}_{opt}=\left[\frac{N}{{d}_{max}\sqrt{3\rho }}+0.5\right].$$

In this formula, N is the total number of available nodes and is the node density in the CRSN. Furthermore, *d*_max_ is the maximum distance at which data may be sent between CRSN nodes.

Cluster Heads (CHs) are chosen at random from the clusters that are in communication range of the base station^21^. In order to get data from the base station to the other nodes in the CRSN, a set of CHs must be chosen. When calculating its distance from the CH nodes, each CRSN node plays an essential role. The CHs are ranked using a least-cost fitness function using the proposed AACDIC algorithm. This method reduces the time needed for the CRSN to converge and increases the amount of time its energy may be used effectively. These very effective clusters help with channel allocation on the fly.

Primary users lose access to channels within their transmission range after Secondary Users (SUs) have assigned them (PUs). Opportunistic spectrum access, which is often used in next-generation networks, is consistent with this method of dynamic spectrum allocation. In this setup, SUs may communicate and exchange locally detected data with one another autonomously. In a distributed cooperative sensing setting, all cognitive users independently locate principal channels that are not in use.

Spectrum management is the process by which the properties of the spectrum, the capabilities of CR reconfiguration, and the needs of end users are taken into account to choose the optimal spectral band for communication. The total quality of service (QoS) is improved by this method of selecting the appropriate spectral band. To avoid congestion, SUs and PUs reserve free channels when they are recognised. Each PU has its own colour code, which corresponds to the channel it belongs to. Within CRSN nodes or within the same cluster, PU nodes do not use common channels.

With the help of its associated channels, each CH determines which of the available channels best represents it. Cluster heads perform condition checks during channel selection by choosing channels in close vicinity to PUs. To efficiently solve the difficulties of multimodal optimization, a variety of clustering methods are used to improve communication amongst the top CHs, with channel member assignments determined by reducing node power.

The hypothesis model uses criterion specified in Eq. (2) to find PUs.

2 $$r\left(t\right)=\left\{\begin{array}{cc}n\left(t\right)& {H}_{0}\\ h\left(t\right)+n\left(t\right)& {H}_{1}\end{array}\right..$$

When discussing cognitive radio networks, the signal received by Secondary Users (SUs) is denoted by the equation r(t), whereas the signal broadcast by Primary Users is denoted by the equation s(t) (PUs). Additive White Gaussian Noise (AWGN) with zero mean is denoted by *n(t).* In this context, h represents the channel's amplitude gain.

Additionally, in this statistical paradigm, 'H_0_' represents a spectrum band channel in which there are no Primary Users (PU). The term "null hypothesis" is used to describe this scenario. On the other hand, the presence hypothesis (*H*_*1*_) is used when Primary Users are present on the channel. Spectrum sensing and cognitive radio systems rely heavily on these assumptions to make educated choices regarding channel occupancy and consequent spectrum access.

### Calculations of power using AACDIC algorithm

Each node in a CRSN makes a connection with a Cluster Head (CH) so that it may determine its optimal CH based on its distance from the base station. There are two main requirements for clusters to develop in the CRSN:

The communication range of the base station must be totally contained within the range of the chosen CH, which is a requirement for the first clustering criterion. The CH and the base station will be able to coordinate and communicate efficiently under these circumstances^22^.

The CH chosen for a certain cluster has to have a lot of energy left over, which is why this criterion is so important. To ensure the CH continues to function properly and for as long as it is needed inside the cluster, it is essential that it meet this condition.

Total network communication transmission power is calculated using Eq. (3). The efficiency and effectiveness of the CRSN as a whole are enhanced by this equation's work to optimize power allocation and consumption.

3 $${P}_{tx}=\sum_{k=1}^{K} \sum_{i=1}^{N} {\text{ Dist }}_{min}\left({n}_{i}^{k},{\text{ Center }}^{k}({\text{CH}})\right).$$

An illustration for cooperative AACDIC clustering structure is shown in Fig. 1. The following Equation may be used to determine the shortest possible path between two locations expressed as pairs of coordinates, for example, (x_1_, y_1_) and (x_2_, y_2_) as shown in Eq. (4).

4 $${\text{Dist }}_{min}=\sqrt{{\left({x}_{0}-{x}_{1}\right)}^{2}+{\left({y}_{0}-{y}_{1}\right)}^{2}.}$$

The goal of intra-cluster communication is to transport data from the source node to the central location of the cluster head (CH) over all available channels, hence minimizing the distance over which data must travel to reach the CH. It can be shown that the centre position of the CH for the kth cluster corresponds to the minimal distance for the ith node inside the cluster, and this is represented by Eq. (5).

5 $$\begin{array}{c}{P}_{\text{intra }}( \, {\text{T}}{\text{o}}{\text{t}} \, )=\sum_{k=1}^{K} {\text{ Dist }}_{min}{\left({n}_{i}^{k},{\text{Center}}(CH)\right)}_{{C}_{k}}\\ =\sum_{k=1}^{K} {\text{ Dist }}_{min}\left({n}_{i}^{k},{n}_{j}^{k}\right){c}_{k}\end{array}.$$

Cluster Heads (CHs) play a key role as hubs in inter-cluster communication, taking data from various source nodes and relaying it quickly and effectively to other CHs in close proximity. Finding the shortest line of communication between the grouped nodes is the goal here. Using Eq. (1), we can determine that the central location of the CH for the kth cluster is represented by (x_kc_, y_kc_), and the minimal distance for the jth node inside the kth cluster is designated as 'd_kj_' as shown in (6).

6 $$\begin{array}{c}CH=\sum_{k=1}^{K} \left(\sum_{i\ne j} {\text{ Dist }}_{min}\left({\text{ Center }}^{k}\left({{\text{CH}}}_{i}\right),{\text{ Center }}^{k}\left({{\text{CH}}}_{j}\right)\right)\right){c}_{k}.\\ \end{array}$$

The 'n^k^_i_' variable in these equations denotes the location at the centre of the ith CH in the *kth* cluster, and the *n*^*k*^_*j*_ variable denotes the location at the centre of the* jth* cluster in the *kth* cluster.

Using the coordinates, we can write the equation for the shortest distance as follows. The equation shows that the minimal distance between clusters is equal to the total of the powers needed for both inter and intra-cluster communication (7). Power necessary for inter-cluster communication is denoted by *'P*_*inter*_*'* whereas power needed for intra-cluster communication is denoted by *'P*_*inter*_*'* in this context.

7 $$P\left(\text{ Tot }\right)=\sum_{i=1}^{N} \left({P}_{\text{intra }}+{P}_{\text{inter }}\right){n}_{i}.$$

### Proposed AACDIC algorithm

**Figure 2:**
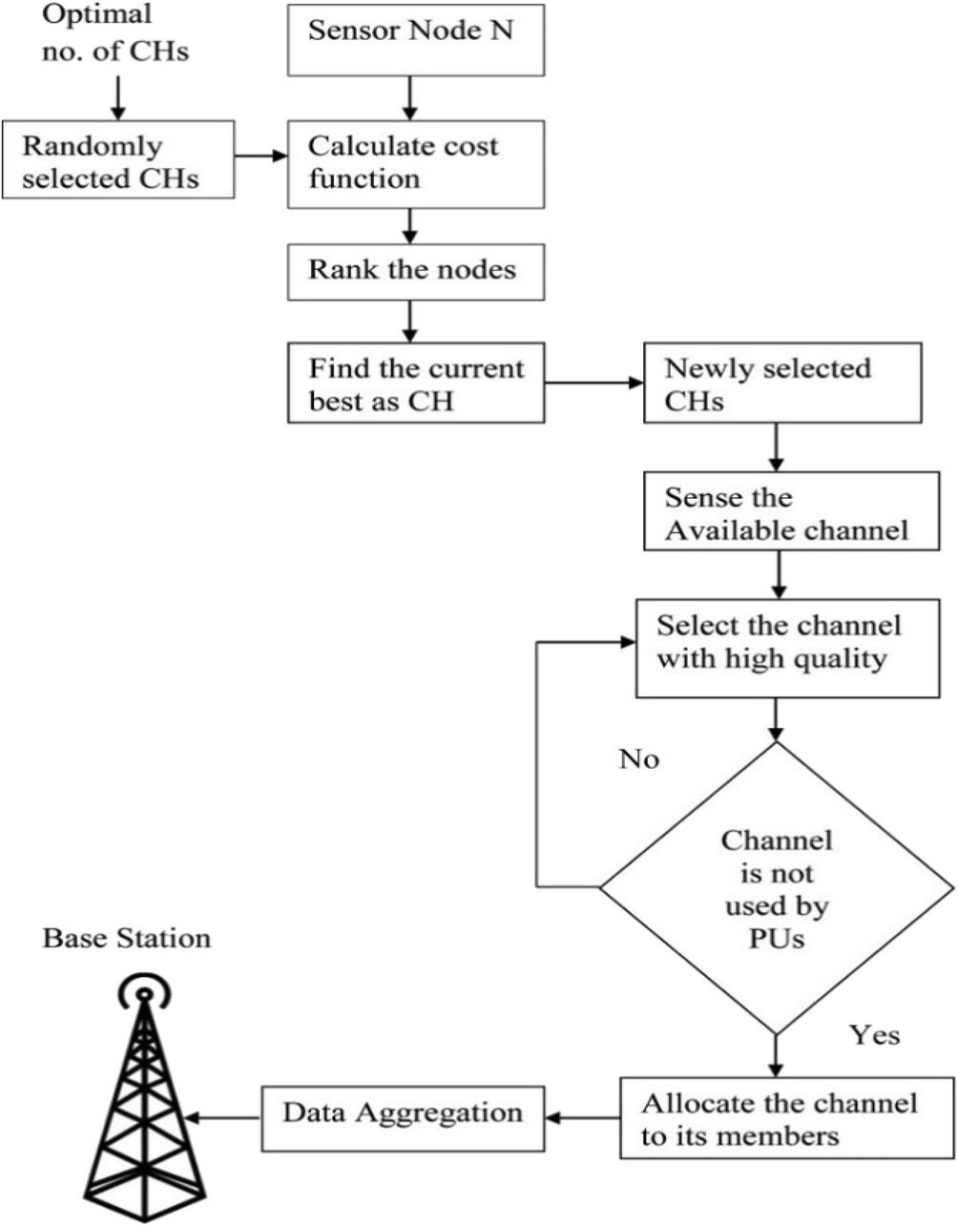
Workflow of AACDIC.

As can be seen in Fig. 2, the proposed AACDIC sensor network consists of 'N' nodes, each of which belongs to one of *K* predefined clusters. Important parts of this network architecture include:

CHs (cluster heads) are chosen by: S is the number of element sets utilised to construct K completely hypothetical CHs, where K is an arbitrary integer. The distance *d(n*_*i*_*, CH*_*p,k*_*)* between each node represented by 'n_i_' (where i varies from 1 to n) and every conceivable CH location is calculated. After then, each node *'n*_*i*_*'* is given a CH point. The notation *'CH*_*p,k*_*'* stands for the *kth* CH linked to particle 'p' while the notation *'d(n*_*i*_*, CH*_*p,k*_*)* = *mind(n*_*i*_*, CH*_*p,k*_*)'* stands for the minimal distance selection criteria, where k may be any value from 1 to k.

Base station acknowledgement centralized algorithm: To aid in the clustering process and provide critical data to the base station, a centralized method is used, as described in Ref. ^16^. The ultimate decision on which CHs to use at the base station relies heavily on an algorithm called FA. In order to guarantee effective cluster formation and network functioning, its major goal is to pinpoint the ideal locations for CHs using a cost function as shown in equations from 8 to 10.

8 $${\text{Cos}}t={f}_{1}\times \beta +{f}_{2}\times \left(1-\beta \right),$$

9 $${f}_{1}=\underset{k-\mathrm{1,2},K}{max} K\left\{{\Sigma }_{\nabla {n}_{i}\in {C}_{p,k}}d\left({n}_{i},C{H}_{p,k}\right)/\left|{C}_{p,k}\right|,\right.$$

10 $${f}_{2}=\frac{\sum_{i=1}^{N} E\left({n}_{i}\right)}{\sum_{k=1}^{K} E\left(C{H}_{p,k}\right)}.$$

The optimal fitness coefficient for a given pair is denoted by "C”. The CHs help determine two important roles by linking K nodes for the cluster particle '*p*’, The Workflow of AACDIC Is shown in Fig. 2.

1. *f*_1_—Max. Avg. dist. This function, labelled '*f*_*1*_,' indicates the CHs chosen for the cluster particle *'p'* with the greatest average distance. It is calculated by measuring the gap between CHs and individual cluster nodes.

The average energy of the CH nodes is represented by the function *f*_*2*_, and for particle 'p' this is expressed as a ratio to the energy of the CH nodes.

The energy at the ith node is denoted by *E(n*_*i*_*),* while the energy at the kth CH node for particle p is denoted by *E(CH*_*p,k*_*).*

The following procedures make up the operating process of the CHs inside the network:

First, CHs choose channels from the pool of those within their communication range.

Channels with the highest quality are selected, with consideration given to which channels are in use by nearby primary users (PUs).

Third, a common channel is used to collect data from cluster members who are connected to CHs.

The last step is for the base station to send the gathered network data to the allotted CHs. Algorithm 1 shows the steps involved in the proposed work.

**Algorithm 1:**
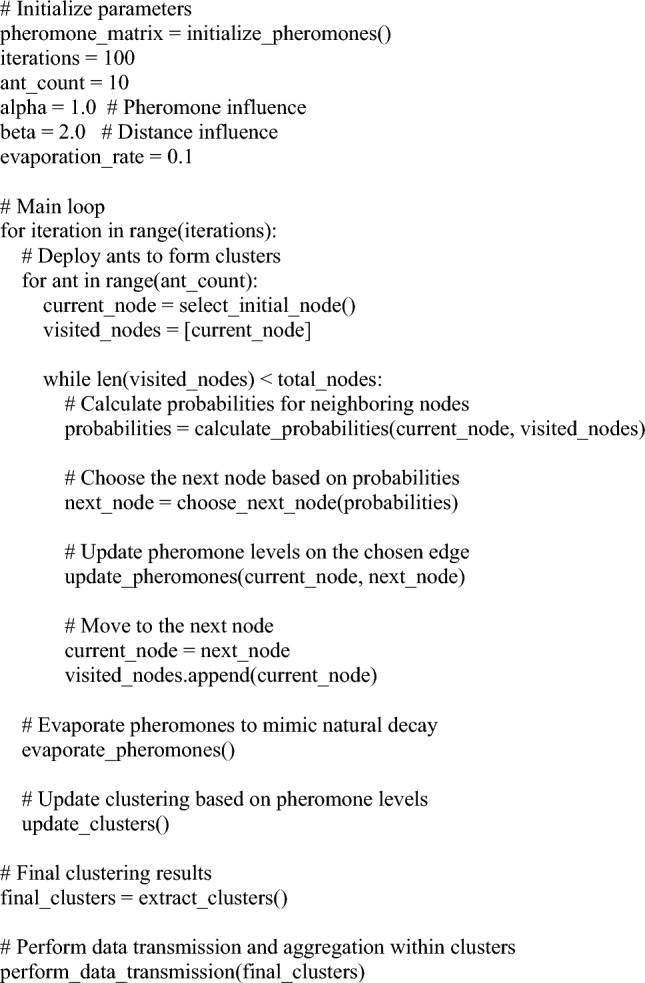
Proposed work algorithm.

This pseudocode provides a high-level overview of the proposed algorithm, incorporating the principles of ant colony optimization for clustering in WSNs. Keep in mind that the actual implementation details, such as how probabilities are calculated, how clusters are formed, and how data transmission/aggregation is performed, would require further elaboration based on the specific characteristics.

## Results and discussions

The performance of the proposed adaptive ant colony distributed intelligent based clustering (AACDIC) in the context of cognitive radio (CR) networks is evaluated in this part using Network Simulator—2 (NS2). For optimal network connection and performance, the assessment seeks to establish the optimum number of clustered structures, K.

**Figure 3:**
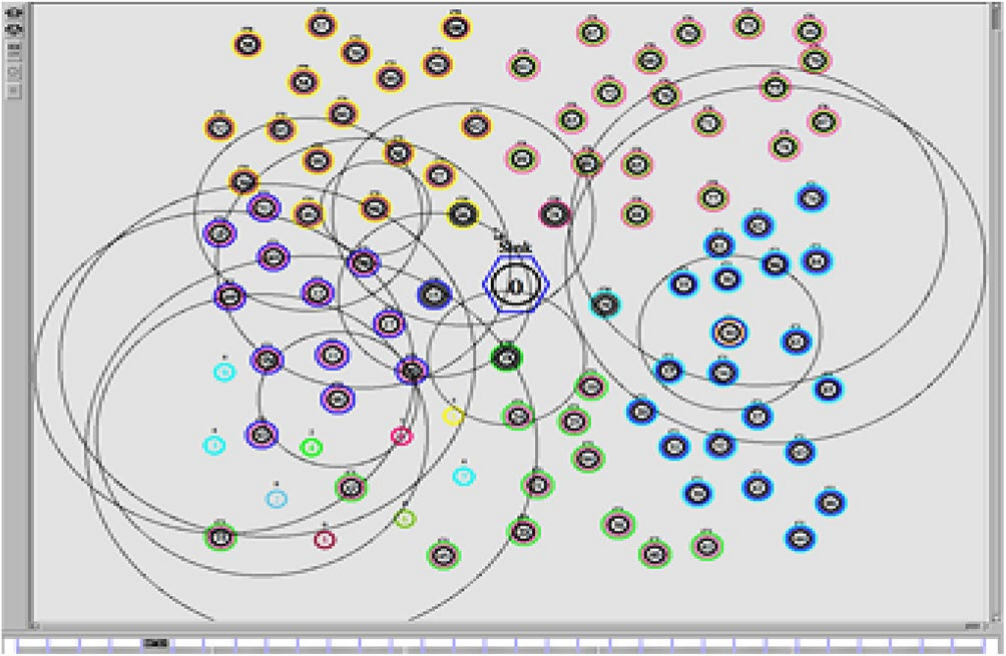
The process of channel distribution for SUs and Pus.

Primary users (PUs) and secondary users (SUs) utilising the available channels in the simulated CR network are summarised in Fig. 3. Ten processing units (PUs) and ninety SU nodes (subscriber units) are used in this simulation to test the efficacy of the dynamic spectrum access concept. These user nodes are spread out throughout a 1000 m by 1000 m area. Ten separate colors—pink, deep pink, blue cyan, sky blue, yellow-green, maroon, yellow, violet red, and green—are used to visually symbolise the ten different channels that may be accessed.

Channels used by SUs are represented by the numbers 0 through 9 in the NS2 simulation (Version 2.34) while channels used by PUs are represented by the numbers 0 through 9. To prevent CRSN neighbours from accessing channels that are already in use by other nodes, each PU chooses from a shared pool of 10 channels, and a protective range of around 200 m is enforced.

The simulation duration used for the study is 131 s, and a constant packet size of 512 bytes is used throughout. Focusing on channel usage, interference mitigation, and network connection, this extensive assessment aims to measure how well the suggested dynamic spectrum access model functions in the context of CR networks.

In this analysis as shown in Fig. 3, we look at how well several suggested advanced clustering algorithms perform in comparison to the current DGCC approach. Conservative merge duration for various cluster-based secondary relay node (CRSN) sizes, conservative node power for various cluster numbers, conservative node power for primary users (PUs) and secondary users (SUs), detection probability, and missed detection with varying probability of false alarm (PFA) values, and detection probability with varying signal-to-noise ratio (SNR) values, are all taken into account during the evaluation.

**Figure 4:**
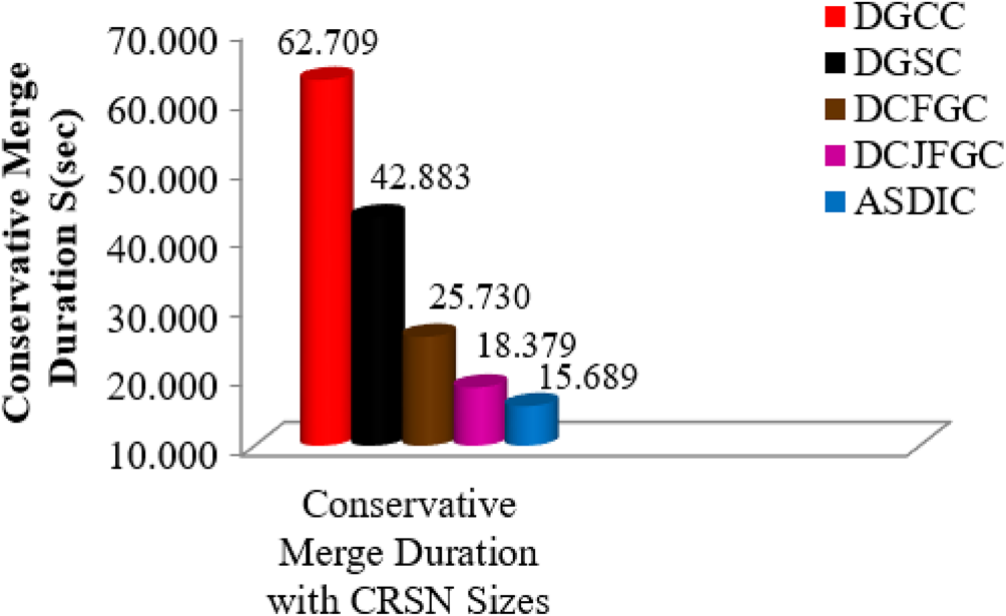
Correlation of conservative merge duration in various improved grouping strategies.

Figure 4 summarizes the conservative merge time for different CRSN sizes using a variety of high-level clustering methods. Figure 4 shows that the proposed AACDIC technique has a much faster Conservative merging time than existing improved clustering algorithms. AACDIC’s Conservative merging procedure is quicker than the current DGCC approach by an average of 47.2 s for a range of CRSN sizes. This finding provides evidence that the AACDIC merges more quickly and effectively, which may boost network performance as a whole.

**Figure 5:**
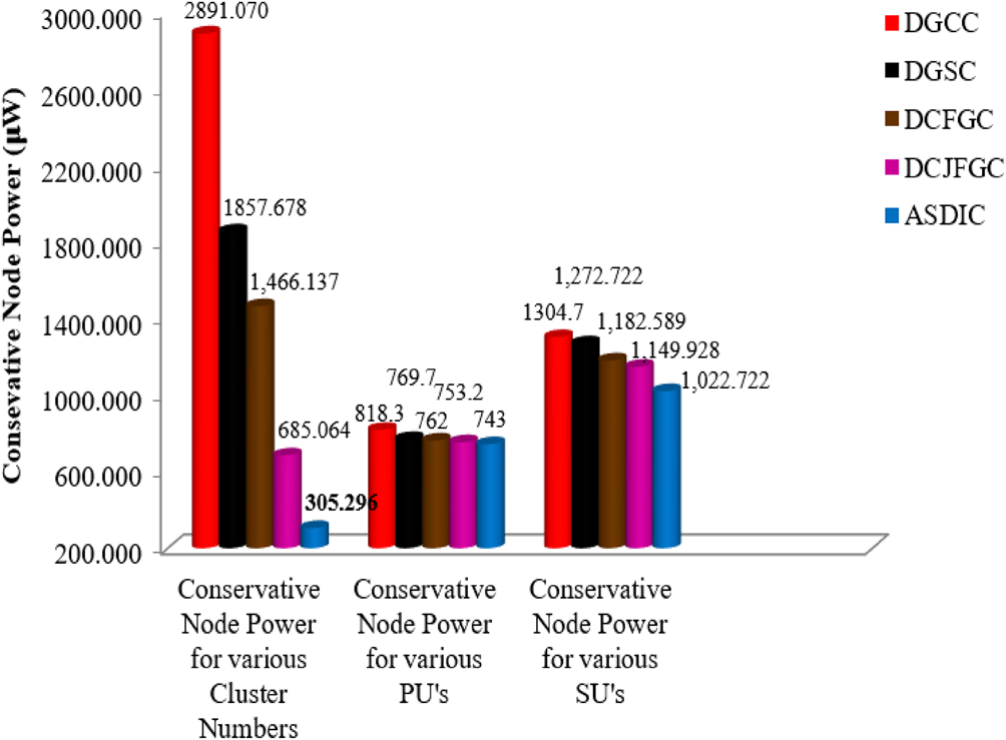
Correlation of conservative node power in various improved grouping strategies.

As can be seen in Fig. 5, one key distinction between the proposed AACDIC and other advanced clustering algorithms is its much reduced conservative node power. In instance, across a variety of cluster topologies, the conservative node power of AACDIC is measured at 2585.774 W, which is lower than that of the conventional DGCC approach.

In particular, the conservative node power for both main and secondary users is lower when comparing AACDIC to other enhanced clustering algorithms. AACDIC delivers a 75.3 W lower conservative node power than DGCC for main users, and a 281.978 W lower conservative node power than DGCC for secondary users.

In Fig. 5, we see a full comparison of node power across several enhanced clustering algorithms, from a PU’s and SU’s perspective. By comparing the AACDIC method with its alternatives, we can see the significant savings in node power usage for both PUs and SUs.

The suggested technique is used to examine the average node power for primary users (PUs) and secondary users, as well as the average convergence time over a range of CRSN sizes (SUs)^23^. In order to gauge performance, we measure the typical energy used by nodes in different cluster sizes. The network's efficiency is profoundly affected by the CRSN's size because of how it interacts with the base station.

When compared to other known optimization clustering methods, AACDIC has a much lower average convergence time. Specifically, while taking into account different CRSN sizes, AACDIC^24^ improves performance by 74.98 percent, with an average convergence time 47.2 s faster than the current DGCC technique.

**Figure 6:**
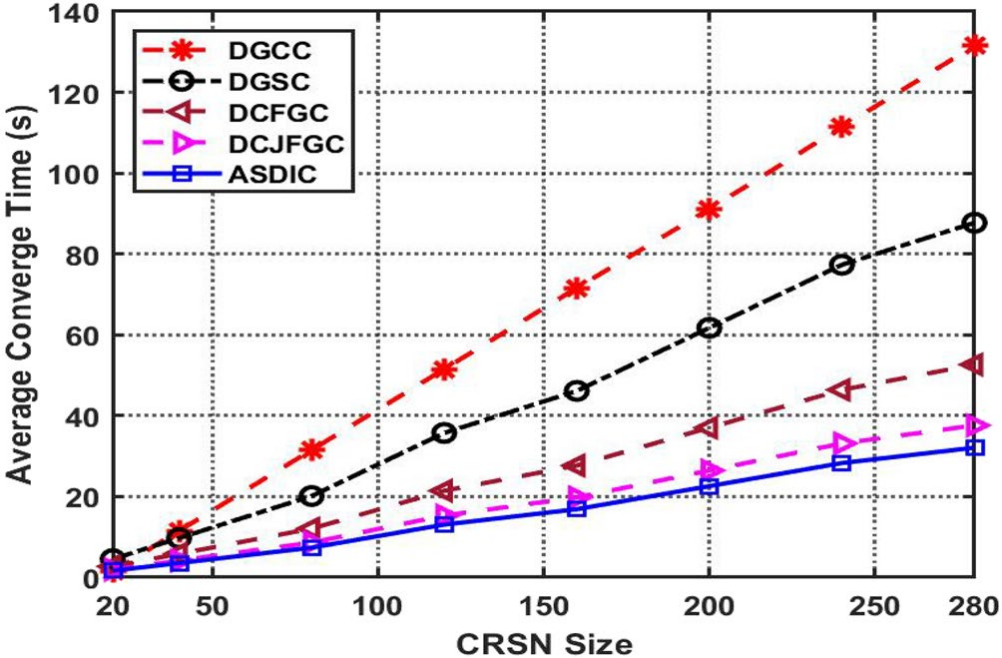
The comparison graph of average convergence time for different CRSN size.

Figure 6 displays the results of a study comparing the average convergence time of several optimum clustering strategies for a range of CRSN sizes. The average time required to converge linearly rises as CRSN size grows. In this study, we estimate a maximum CRSN size of 280, therefore the convergence time^25^ is an important metric for gauging the efficiency of the suggested AACDIC approach.

See Fig. 6 to see how the proposed AACDIC approach compares to other clustering strategies in terms of average node power usage. In particular, AACDIC measures an average node power of 2585.774 W, which is less than that of conventional DGCC across a variety of cluster sizes, leading to an increase in performance of 89.44%.

**Figure 7:**
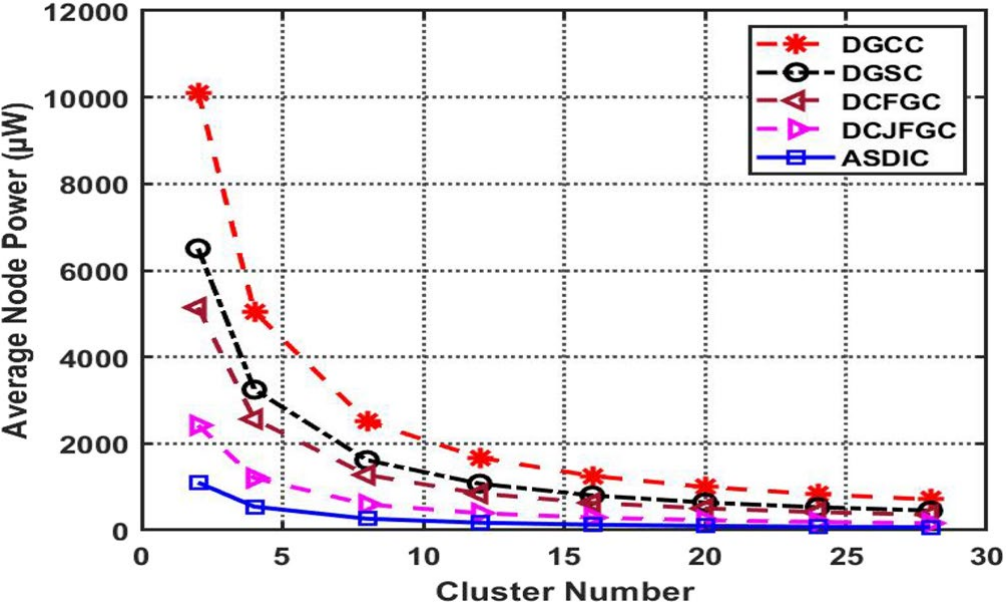
The comparison graph of average nodes power for different cluster number of clusters.

A comparison of average node power across cluster sizes and optimum clustering methods is shown in Fig. 7. From 2 clusters up to 28 clusters, power usage is shown graphically.

By dividing the total energy used by all SUs and PUs in a cluster by the total number of nodes in that cluster, the average node power (in watts, or W) may be determined using Eq. (11). Power efficiency over a range of network cluster sizes may be evaluated with the use of this statistic^26–28^.

11 $$ Average\,\,Node\,\,Power = \frac{{\sum\nolimits_{i = 1}^{N} {PU_{i} \left( {Energy} \right) + \sum\nolimits_{i = 1}^{N} {SU_{i} \left( {Energy} \right)} } }}{Number\,\,of\,\,nodes\,\,in\,\,clusters}. $$

As seen in Fig. 7, once the size of the clusters reaches 28 the simulation stops and the power usage stays constant. The graphic compares the average node power values across several clustering methods, such as the standard DGCC, DGSC, DCFGC, DCJFGC, and the novel AACDIC method.

Using the AACDIC method, the study considers the presence of both Primary Users (PUs) and Secondary Users (SUs) in the network, revealing differences in node-level power usage. As shown in Eq. (1), the mean PU node power is determined by dividing the total energy used by PUs by the total number of PUs in watts (W) as shown in Eq. (12). Learn how the number of clusters created in a network is related to the power consumption of various clustering methods with the assistance of this diagram.

12 $$ PUs\,Node\,\,Power\, = \frac{{\sum\nolimits_{i = 1}^{N} {PU_{i} \left( {Energy} \right)} }}{Number\,\,of\,\,PUs}. $$

**Figure 8:**
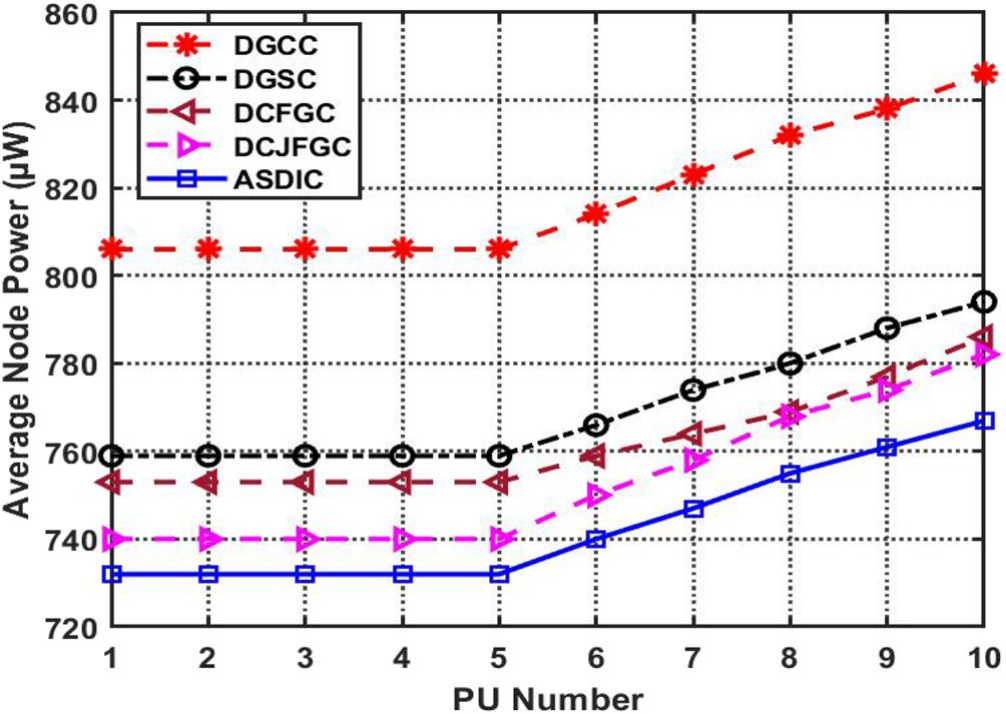
PU’s Average node power with existing optimized clustering approaches.

As the number of primary users grows, so does the average amount of energy used (PUs). Figure 8 shows, for nodes with 10 PUs, how the average power of the cluster as a whole changes as the number of PUs increases, while using the best available clustering optimization methods. This scenario finds AACDIC's average node power for primary users (PUs) to be 75.3 W, which is 9.646 percent lower than that of DGCC.

In Fig. 8, we can see how different optimal clustering techniques affect the average PU power consumption of a node. When more PUs are active within the transmission range, more spectrum is needed for the clustering process. As a consequence, the clustering outcomes are affected by how much power is used. Ten sample Primary User nodes are supposed to be within 200 m of each other in this simulation.

By increasing the efficiency with which the PUs use available spectrum resources while retaining lower average node power consumption, AACDIC highlights its significance among current optimal clustering methodologies. Similar to how we may get the average node power for SUs using Eq. (13).

13 $$ SUs\,\,Node\,\,Power\, = \frac{{\sum\nolimits_{i = 1}^{N} {SU_{i} \left( {Energy} \right)} }}{{Total\,\,Number\,\,of\,\,SU_{s} }}. $$

**Figure 9:**
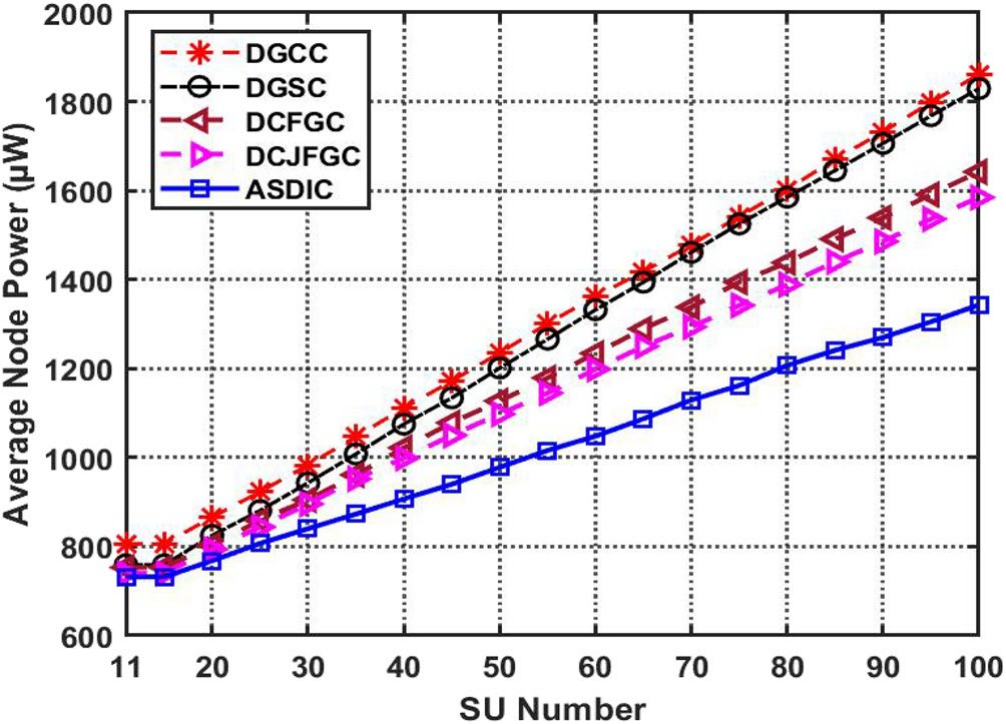
The comparison graph of SUs average node power.

As the strength of the node grows, so does the number of secondary users (SUs). The outcomes of the 90 SUs clustering optimization are shown in Fig. 9. When compared to the average node power for SUs in the current DGCC method, AACDIC's secondary users have been shown to need 281.978 W on average. This analysis shows that AACDIC is superior to other optimal clustering methods in terms of the average power of nodes across all SUs. Ninety auxiliary user nodes are set up within a radius of 150 m of the virtual fortress during the simulation.

By maximizing spectrum usage while reducing secondary users' average node power consumption, the proposed AACDIC methodology outperforms other optimal clustering algorithms. With greater detection rates and reduced false alarm rates, singular threshold detectors shine in cooperative spectrum sensing networks. Spectrum sensing performance is measured by looking at things like false alarm rates, detection rates, and so on. Spectrum sensing approaches rely heavily on SNR estimates for the detection of incoming signals and the formulation of responses.

Based on data from experiments and measurements, we settle on a cutoff value of 4 dB.

- The hypothesis is tested using the likelihood of false alarm rate method when the threshold value is greater than the SNR (showing that the PU channel is incorrectly designated as 'H1'). If > SNR, for instance, we'd settle with H = H1|H0.
- The hypothesis is tested using the likelihood of detection method when the threshold value is larger than the SNR (showing that the PU channel is correctly recognised as 'H1'). When > SNR, for instance, we accept H = H1|H1.

Hypothesis is derived using the likelihood of missed detection technique and has to fulfil the criteria SNR when the threshold value shows a lower SNR compared to the PU channels and 'H0' is not identified. We recognize, for instance, that H = H0|H1.

MATLAB R2021a serves as the backbone of the suggested solution. Finding out when Primary Users are on a channel requires comparing the statistical performance output of Y. (PUs). Probability of False Alarm (PFA) is the probability of choosing H_1_ while the true condition is H_0_, and it is calculated as follows: (14).

14 $$ P_{FA} = P\left( {Y > \lambda |H_{0} } \right) = \frac{{\Gamma \left( {m,\lambda /2} \right)}}{\Gamma \left( m \right)}. $$

The probability of detection (PD) indicates the correctly identified chance H_1_ when it is H_1 as shown_ in the Eq. (15).

15 $$ P_{D} = P(Y > \lambda |H_{1} ) = Q_{m} \left( {\sqrt {2\gamma }_{avg} , \sqrt \lambda } \right). $$

where $\lambda$ is threshold of detection, $\Gamma \left( . \right)$ is complete gamma function, $\Gamma \left( {.,.} \right)$ is incomplete gamma function, $\gamma_{avg}$ is average SNR, $Q_{m} \left( \right)$ is general marcum Q-function. Where, m = TW represents the time of bandwidth product. m = 5 is considered for the AACDIC.

The marcumq() function in MATLAB is used to implement Eq. (15), which calculates the probability of detection (PD).

To increase the PD and reduce the PFA is the goal of the probability of missed detection (PMD) metric. Principal user (PU) presence probability (PMD) is the probability that PUs are using the channel but are unable to identify the primary transmission signal.

**Figure 10:**
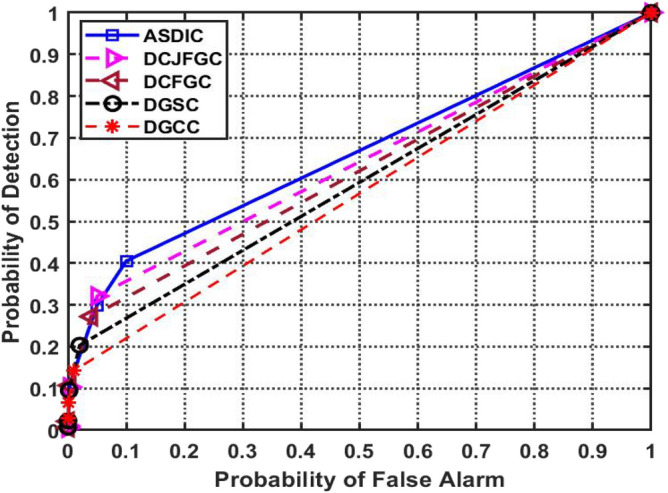
PFA versus probability of detection.

Figure 10 depicts the detection performance with different PFA levels while keeping a 4 dB Signal-to-Noise Ratio (SNR). This SNR is above average and offers sufficient dynamic range for spectral sensing. If the SNR is high, the signal is of excellent quality. The potential for erroneous identification of Primary Users (PUs) as having access to the spectrum bands by Secondary Users (SUs) results in lost possibilities for effective channel use.

In contrast to other methods, the current DGCC approach has a reduced detection probability at low false alarm rates (PFA 0.1). In contrast, the suggested AACDIC model beats the state-of-the-art optimum clustering methods in terms of detection performance when the PFA value is more than 0.1.

**Figure 11:**
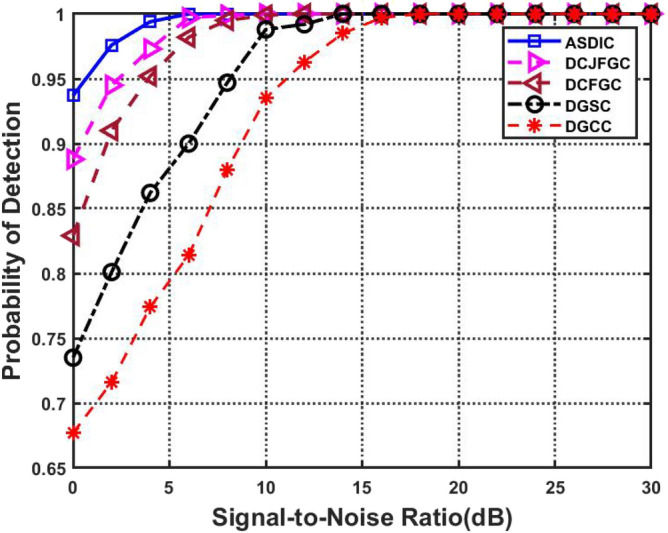
SNR versus PD with optimized clustering from the existing techniques.

With a fixed false alarm probability of 0.1, the detection performance is measured throughout an SNR range of 0–30 dB. The likelihood of detection linearly improves with the SNR until it reaches the maximum value of 1. Figure 11 shows the detection performance at different SNR settings. Less disruption to Primary Users is seen with higher detection probabilities and vice versa.

Compared to the current DGCC approach, which produces a PD of 0.677 at 0 dB SNR, AACDIC's PD of 0.937 is a significant improvement. When compared to other optimal clustering methods, AACDIC's greater performance is shown by the fact that its curve converges to '1' more quickly when the SNR increases above 4 dB.

**Figure 12:**
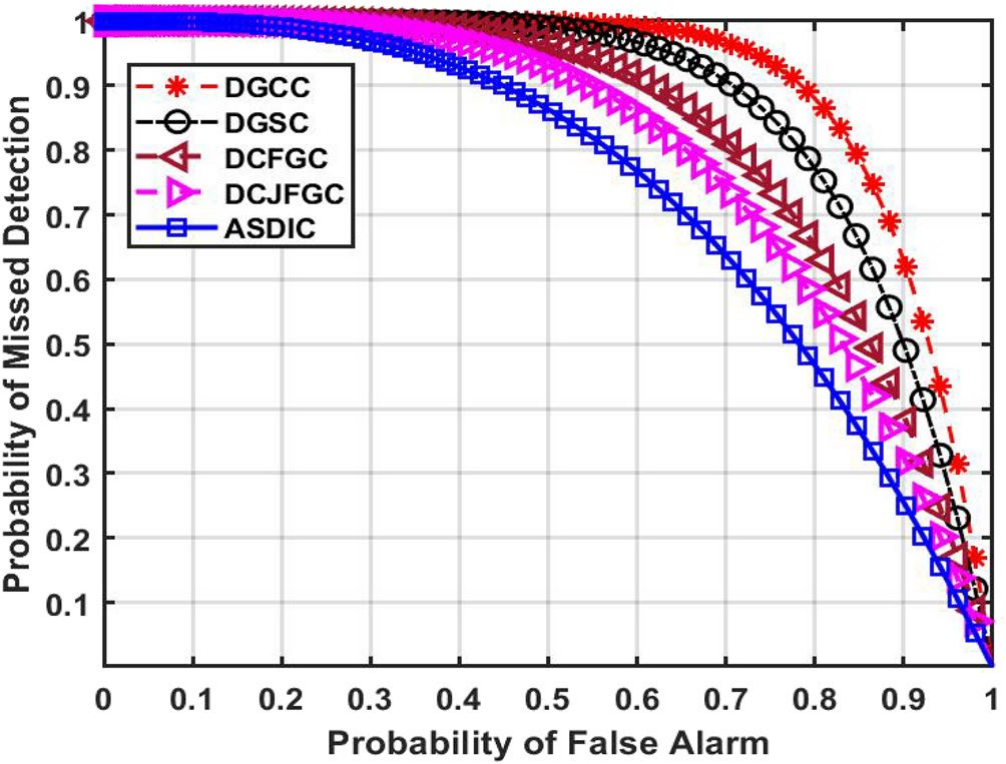
PFA versus missed detection probability.

Figure 12 shows the ROC curve performance of missed detections vs detection with different Probability of False Alarms (PFA). The ROC curve shows that the chance of a missed detection lowers as the PFA rate rises. This suggests that Primary Users (PUs) could be transmitting signals, but that they aren't always being properly recognised as such.

Based on the current DGCC model, the PFA value of 0.6 is the beginning point for a linear increase in the Probability of Missed Detection (PMD) all the way to 1.

When using the suggested AACDIC method, however, the PMD may be as high as 1 even with a PFA of 0.1, and it can drop to 0 as the PFA approaches 1. This shows that, relative to previous optimization procedures, AACDIC performs remarkably well in detecting main transmissions, even at the greatest false alarm rate.

In addition, AACDIC is evaluated with four other leading clustering strategies. These strategies aim to reduce energy consumption in the transmission of data over shorter distances, improve energy efficiency in clusters, and restrict disruptions to the network's key users. In this evaluation, AACDIC outperforms the other methods by a wide margin. Its PD value is 0.372, its PFA value is 0.7, and its PMD value is 0.628.

## Conclusions

In conclusion, our study presents a comprehensive evaluation of the AACDIC Algorithm's performance in wireless sensor networks (WSNs) and demonstrates notable improvements over existing schemes. The results obtained through rigorous simulations and comparisons showcase the algorithm's efficacy in optimizing network efficiency, particularly in mitigating the frequent Cluster Head (CH) re-elections that have posed a persistent challenge in prior clustering algorithms.

The adaptive CH re-election mechanism introduced in the AACDIC Algorithm dynamically adjusts re-election frequency based on real-time network conditions. This adaptability significantly reduces the overhead associated with frequent CH re-elections, promoting stability and extending the operational lifespan of the WSN. The energy-aware criteria incorporated in CH selection further contribute to enhanced energy efficiency, ensuring that selected CHs exhibit a higher likelihood of sustained performance.

Comparisons with traditional clustering algorithms reveal a marked improvement in the AACDIC Algorithm's ability to maintain network stability under varying conditions. The algorithm's adaptability to changing energy levels, communication traffic, and historical CH performance positions it as a robust solution for optimizing WSN performance.

Moreover, our study introduces a novel dimension by considering the impact of Signal-to-Noise Ratio (SNR) on data fusion reliability. The AACDIC Algorithm leverages SNR information to adaptively adjust the data fusion process, enhancing the accuracy and reliability of information transmitted within the network.

In summary, the AACDIC Algorithm presents a holistic solution addressing drawbacks in existing clustering schemes, delivering improved stability, energy efficiency, and adaptability. The positive outcomes demonstrated in our comparative analyses underscore the algorithm's potential as a valuable contribution to the advancement of WSNs, offering a more resilient and efficient approach to clustering and data aggregation in dynamic environments.

## Data Availability

The data used for the findings will be shared by the corresponding author upon request.
